# Nitrile Hydratase Genes Are Present in Multiple Eukaryotic Supergroups

**DOI:** 10.1371/journal.pone.0032867

**Published:** 2012-04-10

**Authors:** Alan O. Marron, Michael Akam, Giselle Walker

**Affiliations:** 1 Department of Zoology, University of Cambridge, Cambridge, United Kingdom; 2 Department of Botany, University of Otago, Dunedin, New Zealand; East Carolina University, United States of America

## Abstract

**Background:**

Nitrile hydratases are enzymes involved in the conversion of nitrile-containing compounds into ammonia and organic acids. Although they are widespread in prokaryotes, nitrile hydratases have only been reported in two eukaryotes: the choanoflagellate *Monosiga brevicollis* and the stramenopile *Aureococcus anophagefferens*. The nitrile hydratase gene in *M. brevicollis* was believed to have arisen by lateral gene transfer from a prokaryote, and is a fusion of beta and alpha nitrile hydratase subunits. Only the alpha subunit has been reported in *A. anophagefferens*.

**Methodology/Principal Findings:**

Here we report the detection of nitrile hydratase genes in five eukaryotic supergroups: opisthokonts, amoebozoa, archaeplastids, CCTH and SAR. Beta-alpha subunit fusion genes are found in the choanoflagellates, ichthyosporeans, apusozoans, haptophytes, rhizarians and stramenopiles, and potentially also in the amoebozoans. An individual alpha subunit is found in a dinoflagellate and an individual beta subunit is found in a haptophyte. Phylogenetic analyses recover a clade of eukaryotic-type nitrile hydratases in the Opisthokonta, Amoebozoa, SAR and CCTH; this is supported by analyses of introns and gene architecture. Two nitrile hydratase sequences from an animal and a plant resolve in the prokaryotic nitrile hydratase clade.

**Conclusions/Significance:**

The evidence presented here demonstrates that nitrile hydratase genes are present in multiple eukaryotic supergroups, suggesting that a subunit fusion gene was present in the last common ancestor of all eukaryotes. The absence of nitrile hydratase from several sequenced species indicates that subunits were lost in multiple eukaryotic taxa. The presence of nitrile hydratases in many other eukaryotic groups is unresolved due to insufficient data and taxon sampling. The retention and expression of the gene in distantly related eukaryotic species suggests that it plays an important metabolic role. The novel family of eukaryotic nitrile hydratases presented in this paper represents a promising candidate for research into their molecular biology and possible biotechnological applications.

## Introduction

Nitrile-containing compounds are widespread in the environment, mainly in plant-derived substances such as cyanoglycosides and cyanolipids [1]. Nitrile hydratases are enzymes that catalyze the hydrolysis of nitriles, converting them into their corresponding amides. Together with amidase enzymes, nitrile hydratases are a component of the biochemical pathway producing ammonia and organic acids from nitrile-containing compounds [2]. Prokaryotic nitrile hydratases comprise two subunits (alpha and beta), encoded by separate genes. The alpha subunit possesses a conserved metal (iron or cobalt) binding domain. The metallic ion ligand is believed to play a crucial role in the catalytic reaction, but the precise nature of the catalysis remains unresolved [3].

### Prokaryotic Nitrile Hydratases

Nitrile hydratases are widespread among the prokaryotes, with the enzyme being found in genera in the Proteobacteria, Actinobacteria, Cyanobacteria and Firmicutes [4]. Here the nitrile-degrading biochemical pathway allows organisms to use nitriles as a nutritional source for obtaining nitrogen (in the form of ammonia) and carbon (in the form of organic acids). Prokaryotic nitrile hydratases and nitrile-related biochemistry have also been adapted for biotechnological roles. Uses include the bioremediation of toxic nitrile-containing compounds (such as herbicides or industrial effluents) from soil and aquatic environments [1], and the industrial synthesis of acrylamide, nicotinamide and 5-cyanovaleramide [5]. The economic importance of nitrile hydratases has led to increased research into their metabolic functions, mechanisms and phylogenetic distribution [3].

### Previously-Known Eukaryotic Nitrile Hydratases

Although prokaryotic nitrile hydratases are well known, prior to this work nitrile hydratase genes had been described in only two distantly related eukaryotic species, *Monosiga brevicollis* [4] and *Aurecococcus anophagefferens* [6].

The choanoflagellates, to which *Monosiga brevicollis* belongs, are confidently placed within the opisthokonts, the eukaryotic supergroup containing the animals, fungi and several unicellular groups such as ichthyosporeans [7], [8]. Genomic and metagenomic analysis by Foerstner and co-workers [4] identified a nitrile hydratase sequence, containing both alpha and beta subunits, in the genome of *M. brevicollis*. Comparison of genome and EST sequences found a 96bp intron present in the sequence, supporting its eukaryotic origin and providing the first evidence of a eukaryotic nitrile hydratase. Phylogenetic analysis of the *M. brevicollis* sequence suggested that it may have originated by lateral gene transfer (LGT) from a proteobacterial species. Additional genomic analyses have found evidence for widespread lateral gene transfer into the *M. brevicollis* genome [9]–[11] a phenomenon believed to be facilitated by its phagotrophic feeding method [12], [13].

*Aureococcus anophagefferens* is a pelagophyte brown alga, a member of the stramenopiles [14]. The analysis of Gobler and colleagues [6] found a nitrile hydratase gene in this alga, but no homologous genes in other stramenopiles. This gene encodes the alpha subunit of the nitrile hydratase protein, and is more similar to the *M.* *brevicollis* nitrile hydratase than to prokaryotic versions of the enzyme. The presence of nitrile hydratase in *A. anophagefferens* was connected to the presence of many other organic nitrogen-degrading enzymes found in its genome by Gobler and colleagues. They hypothesised that the expanded repertoire of nitrogen-related enzymes meant that *A. anophagefferens* can utilize a wider range of organic nitrogen sources, allowing it to out-compete other planktonic species during algal blooms.

### Further Eukaryotic Nitrile Hydratases?

**Figure 1:**
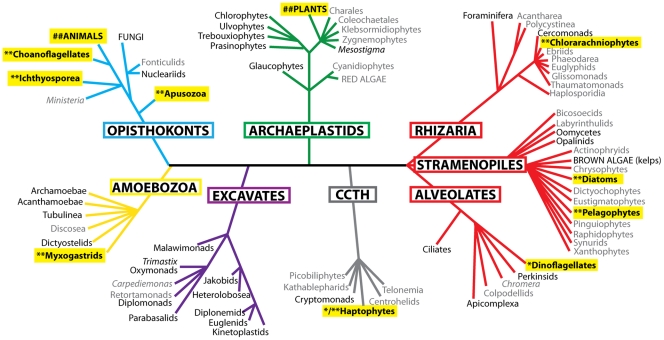
Schematic tree diagram of the eukaryotic supergroups showing the location of nitrile hydratase containing taxa. After Walker *et al.* (2011). Groups labelled in black encompass the taxa listed in table S1; those in grey encompass the taxa in the EMBL/Genbank dbEST, and non-redundant protein and nucleotide databases. Blue = Opisthokonta, Brown = Amoebozoa, Magenta = Excavata, Green = Archaeplastida, Grey = CCTH Supergroup Red = SAR Supergroup, with stramenopile, alveolate and rhizarian labeled. Taxa in capitals contain multicellular species. Taxa highlighted contain species with nitrile hydratase genes. * = eukaryotic-type nitrile hydratase. Number of * indicates the number of subunits present. # = nitrile hydratase subunit genes that may be the result of prokaryotic contamination. Note that taxa branching from a single point represent nodes with ambiguous branching, and the eukaryotic tree is unrooted.

The origin of the eukaryotes was a major landmark in the evolution of life, allowing greater cell complexity, expansion into new ecological niches and the evolution of complex multicellular organisms [15]–[17].The advent of molecular phylogenetics has radically altered traditional views of the taxonomy and diversity of the eukaryotes. Previous classifications into animals, fungi, plants, algae and protozoa have been replaced by the concept of six main supergroups [18], [19] (see figure 1), these being the Opisthkonta, Amoebozoa, Archaeplastida, Excavata, the CCTH supergroup (haptophytes and relatives) and the SAR supergroup (comprising the stramenopiles, rhizarians and alveolates). Phylogenetic analyses unite the opisthokonts and the amoebozoans, which are referred to together as unikonts. The archaeplastids (comprising land plants, green algae and red algae), the SAR supergroup and the CCTH supergroup are often grouped together; with the excavates distinct from this clade and the unikonts. However, it is important to note that the rooting of the eukaryotic phylogenetic tree is unresolved, meaning that the branching order of the major groups remains unknown (reviewed in [19]).

Notwithstanding current debate about branching orders, the two eukaryotic lineages containing nitrile hydratase genes, the opisthokonts and stramenopiles are highly divergent: their last common ancestor is likely to have lived over 1.2 billion years ago and may have been ancestral to all extant eukaryotic groups (see figure 1, [20], [21]). Therefore, the question arises as to whether these eukaryotic nitrile hydratases originated from two separate prokaryote-to-eukaryote LGT events, from a single prokaryote-to-eukaryote LGT event followed by a stramenopile-opisthokont LGT event, or whether nitrile hydratase genes were present in the last common ancestor of the opisthokonts and stramenopiles.

In this paper we provide the results of a wide sampling of eukaryotic taxa (see figure 1). We have found evidence for a characteristic eukaryote-specific nitrile hydratase in the unicellular opisthokonts, the stramenopiles *A. anophagefferens* and *Fragilariopsis cylindrus*, the rhizarian *Bigelowiella natans* and in the haptophyte *Emiliania huxleyi*. This gene comprises a fusion of the beta and alpha subunits, with the beta subunit 5′ of the alpha subunit. At least partial nitrile hydratase genes containing subunits most similar to the eukaryote-type fusion sequences were also found in the amoebozoan slime mould *Physarum polycephalum*, the alveolate dinoflagellate *Karenia brevis*, and the haptophyte *Isochrysis galbana*. This leads to the conclusion that nitrile hydratase was present in the last common ancestor of all eukaryotes, but has undergone multiple losses, notably in the animals and fungi, and possibly also in land plants.

## Results

### Database Searches, Intron Analyses and Protein Domain Analyses

BLASTp and tBLASTn searches of the Broad Institute Origins of Multicellularity website using the *M. brevicollis* nitrile hydratase protein sequence found similar sequences in the genomes of *Salpingoeca rosetta*, *Thecamonas trahens* and *Sphaeroforma arctica* (see table S2). Each genome contained only one significant hit, indicating that no other paralogous genes were present. All hits were also recovered from transcriptome data, indicating that these represent expressed genes. Both alpha and beta subunit protein domains were present fused together in all three gene sequences (see table S2). No introns were found in the *T. trahens* gene. The *S*. *rosetta* gene has three introns and the *S. arctica* gene has 10 introns. None of these introns found are present in the same position as the single intron found in the *M. brevicollis* nitrile hydratase gene [4].

Searches of an EST dataset from the loricate choanoflagellate *Stephanoeca diplocostata* (Marron *et al.* in preparation) revealed the presence of one contig with significant similarity to *M. brevicollis* nitrile hydratase. To confirm that the assembled contig from the EST dataset corresponded to a single gene present in the cultures, primers were designed to suitable regions at the 5′ and 3′ ends of the contig. RT-PCR on cDNA synthesized from total culture RNA amplified a product 996bp in size (EMBL/Genbank accession number FR822187), which matched the EST contig sequence (data not shown). The amplified sequence contained both alpha and beta subunits (see table S2).

In order to determine that the gene is derived from *S.* *diplocostata*, and not from a prokaryotic contaminant, we modified the PCR conditions to locate any introns that may be present in the genomic DNA sequence. A third primer was designed and culture genomic DNA (gDNA) used as template. This PCR amplified a product 356bp in size (EMBL/Genbank accession number FR822186), 236bp longer than predicted from the cDNA sequence. ClustalX alignment of cDNA derived and gDNA derived sequences found a single 236bp stretch present in the gDNA sequence but absent in the cDNA sequence. This stretch was flanked by two regions of perfect alignment, confirming the presence of an intron. PCR using culture gDNA template and the outermost primer pair failed to amplify any relevant products, even under protocols designed to recover longer sequences, suggesting the presence of additional introns.

Searching the EMBL/Genbank protein database for *Aureococcus anophagefferens* proteins using the *M. brevicollis* nitrile hydratase sequence recovered the previously detected nitrile hydratase alpha protein (accession number EGB09110, [6]). However, tBLASTn searches of the *M. brevicollis* sequence against the EMBL/Genbank *A. anophagefferens* WGS dataset found a significant hit that aligned to the full length *M. brevicollis* protein, *i.e.* both alpha and beta subunits (see table S2). The genome scaffold producing this hit was downloaded from the JGI *A. anophagefferens* genome database and translated to find the open reading frame (ORF) matching the known nitrile hydratase alpha protein sequence. Protein domain analysis found both alpha and beta subunits in the same fusion architecture seen in the opisthokont nitrile hydratases (see table S2). A tBLASTn search of the *A. anophagefferens* transcriptome database identified overlapping ESTs corresponding to the nitrile hydratase domains detected by InterProScan. Combining these EST clusters provided the full *A. anophagefferens* nitrile hydratase coding sequence. Two regions of the genomic scaffold were not covered by any EST cluster, and these were taken to represent intronic regions.

Searches of genome databases covering the range of eukaryotic supergroups (see methods and table S1 for details) found significant hits from *Fragilariopsis cylindrus*, *Emiliania huxleyi* and *Bigelowiella natans* (see table S2). No putative orthologs were detected in *B. natans* or *F. cylindrus*. The *E. huxleyi* genome assembly contains several possible orthologs, however only one sequence was found in the consensus EST dataset. This may indicate that *E.* *huxleyi* possesses multiple nitrile hydratase copies that have degenerated into pseudogenes. By comparison to transcriptome data, the *F. cylindrus* gene contains no introns and the *E. huxleyi* gene contains five introns, while the *B. natans* gene contains 17 introns. InterProScan analysis of the *F. cylindrus, B. natans* and *E.* *huxleyi* sequences found both nitrile hydratase subunit protein domains (see table S2).

A tBLASTn search of the *Physarum polycephalum* genomic supercontig dataset found a sequence significantly similar to the alpha subunit region of the *M. brevicollis* protein sequence. Searching *P. polycephalum* EST data recovered several hits aligning to the beta subunit region of the *M. brevicollis* protein sequence (see table S2). These predicted subunit identities were supported by InterProScan analysis results (see table S2). When the *P. polycephalum* genomic supercontig dataset was searched with the EST sequence using tBLASTx, the best hit was to the same supercontig as the best hit to the *M. brevicollis* sequence. The overall gene architecture of the genome supercontig is 5′-beta-alpha-3′, however the incomplete nature of the draft *P. polycephalum* genome means that this supercontig has large regions of missing data. Therefore it was not possible to determine the intron composition of this gene or the full nitrile hydratase coding or protein sequence.

The genome of *Ricinus communis* produced two hits, each to distinct regions of the *M. brevicollis* nitrile hydratase. InterProScan analysis found that one hit contained the nitrile hydratase beta subunit protein domain while the other hit contained the nitrile hydratase alpha subunit protein domain (see table S2). Both hits corresponded to transcript sequences from the *R. communis* transcriptome dataset and are found on the same genomic scaffold. The amino acid sequences corresponding to each subunit are found in different ORFs of this scaffold. Each has start and stop codons within the mRNA sequences, meaning that these subunits are encoded by separate genes, rather than as a fusion gene. No introns were detected in either the alpha or beta subunit gene. tBLASTx searches for each gene against the EMBL/Genbank non-redundant nucleotide database found that the sequences had highest similarity to prokaryotic nitrile hydratase genes.

The *Daphnia pulex* genome produced two different sequences of significant similarity to *M. brevicollis* nitrile hydratase. As with *R. communis*, each hit aligned with distinct regions of the *M. brevicollis* sequence, however unlike in *R. communis* these hits were found on separate scaffolds. InterProScan analysis identified one as possessing a beta subunit domain while the other contains an alpha subunit domain (see table S2). Neither sequence was found in *D. pulex* EST datasets; therefore no introns could be identified. tBLASTx searches for each sequence against the EMBL/Genbank non-redundant nucleotide database found that the most significant hits were to prokaryotic nitrile hydratases, with markedly worse E values for eukaryotic nitrile hydratase hits (data not shown).

Searches of EST databases (see table S1) using the *M. brevicollis* nitrile hydratase protein sequence recovered additional hits to sequences from *Isochrysis galbana* and *Karenia brevis* (see table S2). Protein domain analyses found a nitrile hydratase beta subunit domain in the *I. galbana* sequence and an alpha subunit domain in the *K. brevis* sequence. tBLASTx searches of both EST sequences against the EMBL/Genbank non-redundant nucleotide database found highest similarity to *M. brevicollis* nitrile hydratase, with markedly lower E values for hits to prokaryotic sequences (data not shown). The intron content of these genes could not be determined due to the lack of complementary genomic data.

Two BLAST search results were taken to be erroneous, a *Danio rerio* DNA sequence and an EST hit from *Triticum aestivum* (see table S2). The *D. rerio* sequence was not found in the most recent genome release and was not considered further. The EST sequence from *T. aestivum* gives significant tBLASTx hits to nitrile hydratase alpha subunits from various prokaryotes and *R. communis* (data not shown). The ORF that corresponds to these sequence alignments contains multiple stop codons however, and so this EST sequence was not considered reliable enough to include in further analyses.

Prokaryotic sequences deposited in the EMBL/Genbank database were also searched for genes that encode both subunits as a fusion protein. One nucleotide sequence from the eubacterium *Rhodococcus rhodochrous* was found to contain the coding sequences for both alpha and beta subunits, and this was arranged with the beta subunit 5′ of the alpha subunit as in the *M. brevicollis* nitrile hydratase (see table S2). Further analysis revealed that this was not a subunit fusion gene. Instead the subunit sequences were distinct genes with their own individual start and stop codons, with an untranscribed region between them.

**Figure 2:**
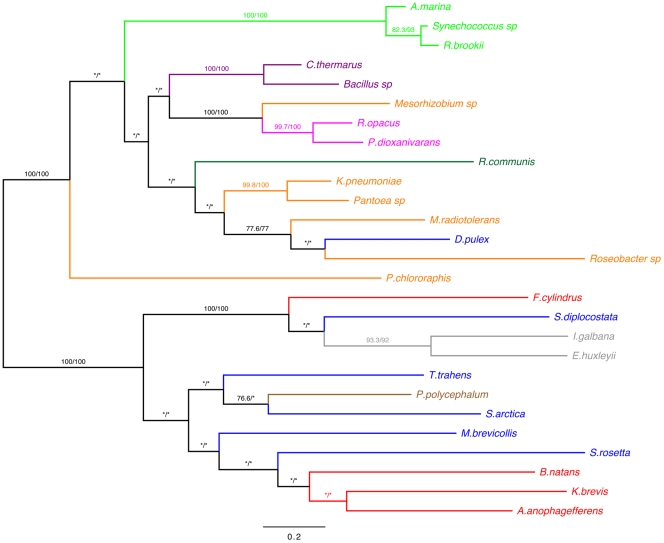
Phylogenetic tree of the concatenated dataset of alpha, beta and fusion nitrile hydratase proteins. The tree was produced using maximum likelihood with the WAG+G+F+I model from an alignment of 658 positions. Numbers at nodes are bootstrap support percentages from PhyML (1000 replicates)/RaxML (100 replicates). Bootstrap values <70 are shown as *. The scale bar indicates the average number of amino acid substitutions per site. Eukaryotic Key: Opisthokonta = blue SAR = red CCTH = grey Amoebozoa = brown Archaeplastida = Green. Prokaryotic Key: Actinobacteria = Magenta Cyanobacteria = Light Green Firmicutes = Purple Proteobacteria = Orange.

### Alignment and Phylogenetic Analyses

Alignments of the nitrile hydratase protein sequences found with the BLAST searches were produced by ClustalX. All but one alpha domain sequences found in eukaryotes possessed the cobalt-type active site VCTLCSC [22]. The only exception was the *K. brevis* nitrile hydratase sequence, which possesses a VCTPCSC active site. It may be the case that this enzyme has altered properties, however nitrile hydratase alpha sequences with the normal VCTLCSC active site were also found from other *K. brevis* EST libraries, albeit in shorter sequences with less significant similarity to the *M. brevicollis* nitrile hydratase. This suggests that the VCTPCSC sequence may be a sequencing or assembly artifact.

The phylogeny generated from a total dataset of concatenated alpha and beta subunits (see methods) is shown in figure 2. The tree is drawn with the root at the split between the ‘eukaryotic-type’ and ‘prokaryotic-type’ clades. This split is well supported, with 100% bootstrap support from both PhyML and RaxML analyses, and is also found in the individual subunit phylogenies with strong support (see figures S1 and S2). All of the beta-alpha subunit fusion nitrile hydratases resolve in the eukaryotic clade, together with three single subunit genes from the eukaryotes *I. galbana, K. brevis* and *P. polycephalum*. The CCTH sequences resolve as a monophyletic clade with good (>90%) support. The maximum likelihood tree of the eukaryotes generated with this nitrile hydratase dataset is not congruent with the eukaryotic supergroup phylogeny (see figure 1) and this incongruence is also found in the phylogenetic analysis of the individual nitrile hydratases subunits alone (see figure S3), however the incongruence is poorly statistically supported.

The ‘prokaryotic-type’ clade contains all of the bacterial sequences, plus sequences ostensibly deriving from eukaryotes (a land plant and an animal) that encode distinct subunits, similar to the situation found in prokaryotes. The nitrile hydratases from both *Ricinus communis* and *Daphnia pulex* fall within the prokaryotic clade with high bootstrap support (see figure 2) and also in the phylogenetic analyses conducted on the individual subunits (see figures S1 and S2). The cyanobacterial, firmicute and actinobacterial nitrile hydratases resolve as well-supported monophyletic clades, with the proteobacterial nitrile hydratases paraphyletic. However as with the eukaryotic clade, the internal branching order of the prokaryotic nitrile hydratase clade is poorly supported by bootstrapping.

Figure 1 shows the distribution of nitrile hydratases across the eukaryotes. Using the results of the phylogenetic analyses, these nitrile hydratase genes can be separated into ‘eukaryotic’ (marked with asterisks) and ‘prokaryotic’ (marked with hashes) clades.

## Discussion

The data presented here provides evidence for a beta-alpha subunit fusion type nitrile hydratase protein in three eukaryotic supergroups; opisthokonts, CCTH supergroup (haptophytes) and the SAR supergroup (stramenopiles and rhizarians). There is further evidence for the presence of both alpha and beta subunits in a possible fusion architecture in amoebozoans. In addition, the nitrile hydratase alpha subunit is present in a dinoflagellate (alveolate) within the SAR supergroup. Both alpha and beta subunits are present in the archaeplastid *R. communis* and the opisthokont metazoan *D. pulex*. The absence of introns, lack of transciptomic confirmation (in *D. pulex*) and the bacterial phylogenetic affinity means that these genes may be derived from prokaryotic contamination.

### Gene Architecture

**Figure 3:**
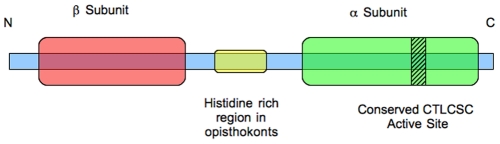
Schematic diagram of the general architecture of the beta-alpha subunit fusion nitrile hydratase found in eukaryotes. This form is known to be found in *M. brevicollis, S. rosetta, S. diplocostata, S. arctica, T. trahens, A. anophagefferens, F. cylindrus, B. natans* and *E. huxleyi. The* red area denotes the beta subunit, which is located N-terminally of the alpha subunit (green region). The CTLCSC active site is located in the alpha subunit, as shown by the shaded area. The yellow area denotes the histidine-rich stretch found between the subunit domains in opisthokonts.

The nitrile hydratase genes found in *M. brevicollis*, *A. anophagefferens*, *S. rosetta*, *S. diplocostata*, *S. arctica*, *T. trahens*, *E. huxleyi*, *B. natans* and *F. cylindrus* (and also potentially in *P. polycephalum*) encode both the alpha and beta protein subunits as a single fusion gene. These fusion genes are arranged with the beta subunit at the 5′ end of the gene (the C terminal of the protein) and the alpha subunit at the 3′ end (N terminal) (see figure 3). Between these subunit domains there is a region of variable length to which InterProScan does not assign a protein domain identity (see table S2).

In contrast, the alpha and beta subunits in prokaryotes are encoded by two separate genes [2]. Here alpha and beta subunit genes are often located together with other nitrile metabolism-related genes in a polycisintronic operon [23], [24]. In most bacterial nitrile metabolism operons the gene coding for the alpha subunit occurs closer to the 5′ end than the gene coding for the beta subunit, the reverse of the situation found in the eukaryotic genes. However, in some strains, such as *Rhodococcus rhodochrous* J1, the beta subunit gene is located upstream of the alpha subunit gene [25]–[28].

Where both genomic and EST data are available, the intronic content of the eukaryotic nitrile hydratase genes can be assessed [4]. Subunit fusion nitrile hydratases have between none (*T. trahens*) and 17 (*B. natans*) introns, with at least one intron in the incomplete *S. diplocostata* sequence. The presence of introns within multiple subunit fusion nitrile hydratase genes is clear evidence of their eukaryotic affinity. No two nitrile hydratase genes investigated share intron positions, a situation suggestive of their long evolutionary history across the eukaryotic supergroups represented.

The opisthokont nitrile hydratases possess a histidine-rich stretch located between the beta and alpha subunit domains. This histidine-rich region is prominent in *T. trahens* (12 residues), *S. arctica* (11 residues) and *M. brevicollis* (17 residues), but shorter in *S. diplocostata* (5 residues) and *S. rosetta* (2 residues). The presence of this opisthokont character in the apusozoan *T. trahens* supports the placement of the Apusozoa as the most basal branch of the opisthokonts [29], [30]. Such repeat sequences may play a role in the spacing and co-ordination of the alpha and beta subunit active sites in the nitrile hydratase protein [31]. The spacer sequence of *B. natans* contains four serine-histidine repeats. In *E. huxleyi* the sequence between the subunit domains has repeats of glycine, glutamate, glutamine, valine and arginine. In *A. anophagefferens* this region contains triplet repeats of alanine, glycine and aspartate. The region between the subunit domains in *F. cylindrus* is shorter than in any of the other known subunit fusion nitrile hydratases and does not contain any residues repeated more than twice in succession, or any obvious compositional bias.

### Phylogeny and Origin of Nitrile Hydratase in Eukaryotes

Foerstner and colleagues [4] proposed that nitrile hydratase in the choanoflagellate *M. brevicollis* originated as the result of a lateral gene transfer event from a prokaryotic source. This prokaryotic ancestor was likely to have been a species of prey bacterium, according to hypotheses connecting phagotrophy and lateral gene transfer [12]. For *A. anophagefferens*, no explanation was given for the presence of a nitrile hydratase alpha gene [6].

The evidence given here is for subunit fusion nitrile hydratase genes being present across four eukaryotic supergroups (see figure 1). The characteristic gene architecture (see above) shared between these eukaryotic sequences, and the absence of any such fusion genes from prokaryotic sequence datasets, is evidence that these fusion genes are homologous across the eukaryotes. This is supported by phylogenetic analysis (see figure 2). The clades containing the fusion protein sequences also contain all of the eukaryotic single subunit proteins (except for those from *D. pulex* and *R. communis*, see below). The overall nitrile hydratase phylogeny is incongruent with the eukaryotic supergroup phylogeny (*cf.* figures 1 and 2). It is likely that this is due to restricted taxon sampling and relatively short nitrile hydratase sequences providing only limited phylogenetic information.

Subunit fusion nitrile hydratases are found in widely divergent eukaryotic supergroups. Though the branching order of eukaryotic groups remains unresolved, the consensus is that the CCTH and SAR supergroups diverged early on from the unikont opisthokonts and amoebozoans. Based on the evidence presented here, the simplest explanation is that a beta-alpha subunit fusion nitrile hydratase gene was present in the last common ancestor of these groups, which could be considered equivalent to the last common ancestor of all eukaryotes [19]. This hypothesis assumes that LGT between supergroups is a rare event [32], which is likely to be a valid assumption in this case due to the diversity of species involved, both taxonomically and ecologically [12]. Further genomic information from the eukaryotic diversity is needed to provide a more definitive answer, but the current evidence points towards nitrile hydratase being ancestral to all eukaryotes.

The individual alpha and beta nitrile hydratase genes found in *D. pulex* and *R. communis* are likely the result of contamination. The lack of introns in either gene does not support a eukaryotic affinity, and the gene architecture of the subunits (two subunits being detected but on distinct open reading frames) is unlike other eukaryotic nitrile hydratases. Both sequences fall within the prokaryotic clade of the phylogenies with strong bootstrap support, branching within the proteobacterial nitrile hydratases (see figures 2, S1 and S2). Contamination from bacteria is a common problem in genome and transcriptome sequencing projects and it is the favoured explanation for the identification of nitrile hydratases in *D. pulex* and *R. communis*.

This suggests that the failure to find ‘eukaryotic-type’ nitrile hydratase sequences in the metazoans, fungi and land plants is due to to loss of an ancestral subunit fusion gene. The evidence for loss of nitrile hydratase is particularly strong for the metazoans and fungi, because of the high sampling density of genome sequences in these taxa (see table S1), and the occurrence of nitrile hydratases in closely related groups (the choanoflagellates and apusozoans, [7], [29]). Similarly the relatively high number of land plant genomes and EST projects that are available [33] make it likely that no ‘eukaryotic-type’ nitrile hydratases are present in the embyrophytes (see table S1).

Subunit loss may have occurred in alveolates and some haptophytes, based on the sequences from *I. galbana* (loss of the alpha subunit) and *K. brevis* (loss of the beta subunit). It is important to note that the only available evidence for this is from transcriptomic, rather than genomic, data. Due to the incomplete nature of EST sequencing [34] the presence or absence of the other subunit cannot be definitively resolved. Nitrile hydratase sequences may be absent from other EST datasets, such as those deposited in TbESTdb [35], because of non-expression of the genes. The conditions under which ‘eukaryotic-type’ nitrile hydratases are expressed are unknown, and it may be that many eukaryotic nitrile hydratases have gone undetected due to no, or low-level, expression in culture conditions. In particular this may the case for the excavates, a supergroup where no nitrile hydratases were found but for which most sequences are from parasitic species (*e.g. Leishmania major* [36]). However overall the greatest obstacle to determining the evolutionary history of eukaryotic nitrile hydratases is lack of sequence data from many groups across the eukaryotic phylogeny, such as the non-land plant archaeplastids.

The reasons for the possible loss of one or other or both subunits from the eukaryotic nitrile hydratase fusion gene can only be speculated upon. It may be related to redundancy due to the expansion of the nitrilase gene family, as is found in fungi and land plants [37]–[39]. It should also be noted that ‘eukaryotic-type’ nitrile hydratases were not found in any of the truly multicellular species investigated (animals, fungi, land plants or the kelp *Ectocarpus siliculosus*), though it is present in the colonial *S. rosetta* and aggregating *P. polycephalum* [15], [16]. Determining possible reasons for subunit loss will require information on the metabolic role of eukaryotic nitrile hydratases, in addition to better taxon sampling.

With respect to the origin of the subunit fusion nitrile hydratases, the timing of the hypothetical prokaryote-eukaryote LGT event [4] is shifted from the origin of the choanoflagellates (*c.*1000Ma, [21], [40]) to the origin of the eukaryotes (>1.2Ga, [20], [21]). The presence of nitrile hydratase in the aplastidic unikonts and monophyletic grouping of the cyanobacterial nitrile hydratase subunits (see figure 2) argues against a cyanobacterial origin by plastidic transfer. A proteobacterial source is possible, with the nitrile hydratase genes being imported from the mitochondrion and the fusion of the subunits only happening within the eukaryotic genome; however it is equally possible that the gene was ancestrally nuclear within the eukaryotes.

The additional sequence data presented here makes it unlikely that nitrile hydratase genes were transferred by an LGT event from prokaryotes directly to choanoflagellates, as proposed by Foerstner and colleagues [4]. An origin by LGT has been proposed for several other genes found in choanoflagellates [9]–[11]. It must be noted that while these LGT interpretations are valid based on current evidence, future sequence data may revise these predictions. The possibility of an origin by prokaryote-derived lateral gene transfer remains for the nitrile hydratase genes found in *R. communis* and *D. pulex* (although these genes may be derived from bacterial contamination, see above).

### Molecular Biology of Eukaryotic Nitrile Hydratases

The retention and expression of the nitrile hydratase fusion protein in distantly related eukaryotic groups suggests it may play an important metabolic role in some eukaryotes.

The nitrile degradation pathway requires both nitrile hydratase and amidase enzymes [2]. Amidases are present across the eukaryotes and are evolutionarily distinct from the prokaryotic versions of the enzyme [41], [42]. In addition, the nitrilase enzymes also degrade nitrile-containing compounds and are widespread throughout the eukaryotes [1], [37]. In the eukaryotic genomes where nitrile hydratase has been found, the genes belonging to the nitrilase enzyme superfamily are also present (data not shown). The nitrilase metabolic pathway is shorter than the nitrile hydratase pathway, lacking the need for an amidase step [1]. The repeated losses of nitrile hydratases may be due to it becoming redundant due to the presence of the nitrilase pathway. This may be relevant to the land plants and fungi, both of which possess specialized nitrilase enzymes for the catabolism of cyanide-containing compounds [38], [39].

Foerstner and colleagues [4] were unable to predict the substrate for the *M. brevicollis* nitrile hydratase; however they did postulate that it had a nutritional role. A similar role in the utilization of organic nitrogen was also proposed for the enzyme in *A. anophagefferens* [6]. The identification of nitrile hydratases in additional species means that the enzyme is now found in a wider variety of lifestyles. Nitrile hydratase possessing species are found in aquatic (*e.g.* choanoflagellates, [43]) and terrestrial (*P. polycephalum* [44]) habitats and have a range of nutritional modes, from bacterivorous heterotrophs (*e.g. P. polycephalum*, *T. trahens*) to photoautotrophs (*e.g. A. anophagefferens*, *F. cylindrus*, *E. huxleyi*) to putatively parasitic symbionts (*S. arctica*). The ecological zones of these species vary from tropical waters (*e.g*. *A. anophagefferens* [6]), through temperate climates (*e.g. S. diplocostata* [45]) to arctic waters (*e.g. F. cylindrus* [46]). This ecological diversity makes it unlikely that eukaryotic nitrile hydratases relate specifically to one environmental niche or mode of life.

All of the alpha subunit domains found in eukaryotes (see table S2) possess the cobalt-type active site (VCT), rather than the iron-type active site (VCS). Cobalt-type nitrile hydratases in bacteria have a preference for aromatic nitrile containing compounds [22], but due to the absence of any functional research on the proteins and their enzymatic activity it is unknown if this is the case for eukaryotic versions of the enzyme. Eukaryotic nitrile hydratases may act on a variety of substrates, given that within single bacterial strains there can exist large range of substrate activities [28].

With the increasingly important biotechnological role of prokaryotic nitrile hydratases in topics such as bioremediation and industrial amide production [5], further study of the eukaryotic nitrile hydratase is warranted. It is possible that the reorganization of the gene architecture has proffered higher activity, more efficient regulation or increased stability to the enzyme, as proposed by Foerstner and colleagues [4]. Alternatively the enzyme may have been incorporated into a new metabolic pathway and may have novel biochemistry and substrate specificity compared to prokaryotic nitrile hydratases or eukaryotic nitrilases. This possibility makes the beta-alpha subunit fusion version of the enzyme a promising area for further research.

### Conclusions

Here we report the identification of nitrile hydratase genes from multiple eukaryotic species across four supergroups. These genes are characterised by a specific architecture whereby alpha and beta subunits are fused in a 5′-beta-alpha-3′ orientation. This arrangement of subunit domains, in addition to the results of phylogenetic analyses, is evidence for the homology of these eukaryotic nitrile hydratase genes. The simplest interpretation of these findings is that a nitrile hydratase gene, containing a fusion of both alpha and beta subunits, was present in the last common ancestor of all eukaryotes. Gene loss appears to have occurred in several species, and nitrile hydratase may be completely absent in two supergroups. However, the retention and expression of this gene in multiple species is evidence for it having a metabolic role. Further research into these eukaryotic nitrile hydratases and their function may yield new biotechnological applications for these enzymes.

## Methods

### Database Searches

The nitrile hydratase protein sequence from *Monosiga brevicollis* (EMBL/GenBank GI:167524980) was downloaded and used to search for similar sequences in publicly available genome and transcriptome databases, and the *S. diplocostata* EST dataset (Marron *et al.* in preparation). All databases were searched using tBLASTn and BLASTp with default settings [47] where translated protein sequences were available. The cut-off value for significance was 1E^−05^. In the case of assembled genome databases, sequences producing a significant hit were used to re-query using both BLASTp and tBLASTn to search for any orthologous sequences in the genome. Filtered or masked databases were searched, except in cases where genomic searches recovered one subunit but not the other (*B. natans*). In this case unfiltered databases were queried to search for masked-out subunits associated with the previously recovered genomic sequence.

The list of taxa and non-Genbank databases searched is given in table S1. The EMBL/Genbank non-redundant nucleotide, protein and EST databases were also searched, these names are not included in table S1 as the total number of potentially searched eukaryotic taxa is very high - there are *c.*62,000 taxa with either protein or nucleotide data, with *ca.* 2300 eukaryotes having EST data, and of these 1285 have more than 1000 sequences (dbEST release of December 1, 2011, http://www.ncbi.nlm.nih.gov/dbEST/dbEST_summary.html).

The EMBL/Genbank Eukaryotic Genomes database, and the non-Genbank databases TBestDB, the Broad Institute Origins of Multicellularity Project and Fungal Genome Initiative, Joint Genome Institute databases, Flybase, GiardiaDB, CryptoDB and AmoebaDB: collectively yielded data from 13 amoebozoans, 1 apusozoan, 319 opisthokonts (153 animals, 159 fungi), 27 excavates, 29 archaeplastids (16 embryophytes), 6 members of the CCTH supergroup, and 57 members of the SAR supergroup (33 apicomplexans), as shown in table S1. Most of these, but also considerably more other taxa, were represented in the EBML/Genbank databases that are not listed in table S1.

### Analysis of BLAST Results

The list of BLAST hits with significant similarity to the *M. brevicollis* nitrile hydratase protein sequence is given in table S2.

The hits to opisthokont genome scaffold sequences from the Broad Institute Origins of Multicellularity Project database were checked against the corresponding EST reads to look for evidence of introns.

The genome sequence scaffold from *Aureococcus anophagefferens* that had significant similarity to the full length of the *M. brevicollis* nitrile hydratase (see results) was downloaded from JGI database. Transeq (http://www.ebi.ac.uk/Tools/emboss/transeq/) was used to translate the nucleotide sequence into the ORF containing the nitrile hydratase alpha subunit sequence previously identified [6]. InterProScan [48] (http://www.ebi.ac.uk/Tools/pfa/iprscan/) was used to search for the protein domains present in this translation. The ORF was used in a tBLASTn search against the *A. anophagefferens* EST clusters database. Regions which provided a match an EST cluster sequence were taken to be exonic regions, those regions that did not match to any EST cluster were taken to be introns.

BLAST hits to genomic scaffolds in *E. huxleyi*, *F. cylindrus, B. natans*, *R. communis* (for each subunit individually) and *D. pulex* (for each subunit individually) were checked for the presence of introns by comparison to their transcriptomes. In the case of *B. natans*, the unfiltered transcriptome dataset was used. The corresponding protein sequences from each species were analyzed by InterProScan to determine the number and identity of nitrile hydratase subunit domains present.

tBLASTn searches against the supercontig database of the *Physarum polycephalum* genome found one supercontig with similarity to the *M. brevicollis* nitrile hydratase protein sequence (see results). This hit was analyzed using Transeq and InterProScan to determine the nitrile hydratase subunit domains present. The EST sequence from *P. polycephalum* that gave the most significant tBLASTn hit (see table S2) was similiarly investigated to determine the subunit domains present. This EST sequence translation was used in a tBLASTn search of the *P. polycephalum* supercontig database. The most significant hit was to the same supercontig that gave a significant hit to the *M. brevicollis* sequence and contained the nitrile hydratase alpha subunit domain (see results). The large amount of missing sequence in the subunit containing supercontig (see table S2) meant that determining the full coding sequence and total intronic content was not possible from genomic data, and therefore it cannot be confirmed whether a beta-alpha subunit fusion nitrile hydratase gene is present (see results).

For significant tBLASTn hits from EST databases, Transeq was used to retrieve the full translation that corresponded to the protein alignment to the *M. brevicollis* nitrile hydratase protein. For species with multiple hits, the longest EST hit was used. Species where nitrile hydratases had been previously found in BLAST searches of genomic datasets were ignored, expect in the case of *P. polycephalum* (see above). InterProScan analysis determined the identity of any nitrile hydratase subunit domains present. Reciprocal tBLASTx searches against the EMBL/Genbank non-redundant nucleotide database were performed for sequences containing only one subunit protein domain, in order to investigate for similarity to eukaryotic nitrile hydratases versus prokaryotic enzymes. Greater similarity to prokaryotic enzymes may be indicative of bacterial contamination.

Nitrile hydratase genes containing a fusion of subunits in the 5′-beta-alpha-3′ configuration was searched for using the *M. brevicollis* sequence. tBLASTn searches were conducted against the EMBL/Genbank non-redundant nucleotide database for Archaea, Eubacteria and non-eukaryotic EST datasets.

### *S. Diplocostata* Nitrile Hydratase Amplification and Analysis

In order to confirm that the assembled contig from the EST dataset (Marron *et al.* in preparation) derives from a gene present in *S. diplocostata*, it was necessary to amplify and clone the sequence and demonstrate of the presence of an intron within the gene. Comparison of genome (EMBL/GenBank: ref NW_001865051.1 :399593–401505 Monosiga brevicollis MX1 MONBRscaffold_14 genomic scaffold) and EST (EMBL/GenBank: gi 167524979 ref XM_001746773.1) data from *M. brevicollis* found an intron site around the alpha subunit active site. The equivalent site in the *S. diplocostata* sequence was used to design primer sets that would amplify both the full contig sequence (NitH_F1 5′-GCACTCACGCCTGAGCAGTAT-3′ and NitH_R1 5′-ACGGCACGTGCCCGGTATT-3′) and the region surrounding the predicted intron (NitH_F2 5′-TGGAGAATACGCCTGAAACACACA-3′ and NitH_R1 5′-ACGGCACGTGCCCGGTATT-3′).

gDNA was extracted from *S. diplocostata* cultures using a CTAB buffer based method [49]. RNA was extracted using a Trizol (Invitrogen) based protocol. DNA contamination was removed from the RNA preparation using TURBO DNase (Ambion) with the accompanying buffer and 50mM EDTA as stop solution. The DNA-free RNA was then used for reverse transcription. The cDNA was made using oligo dT primers and Superscript III First-Strand Synthesis reverse transcriptase (Invitrogen).

Both gDNA and cDNA was used as template for both primer combinations. The PCR protocol involved a hot-start denaturing step at 94^o^C for 4 minutes, 35 cycles of 94^o^C for 30 seconds, 56^o^C for 30 seconds and 72^o^C for 75 seconds, with a final extension step at 72^o^C for 5 minutes.

A second method utilizing Expand Long Template Enzyme taq (Roche) was used with gDNA template and the F1+R1 primer combination, to improve amplification of longer intron-containing PCR products. The PCR protocol used in this case was a hot-start denaturing step at 94^o^C for 2 minutes, 35 cycles of 94^o^C for 30 seconds, 56^o^C for 30seconds and 68^o^C for 2 minutes, with a final extension step at 68^o^C for 7 minutes.

PCR products were purified using the QIAquick Gel Extraction Kit (Qiagen) and cloned into the PGEM-T Easy Vector System (Promega) with DH5α Competent Cells (Invitrogen). The plasmids were extracted using a Qiaprep Spin Miniprep Kit (Qiagen). Sequencing was carried out by SourceBioScience (Cambridge, UK).

The sequences returned were assembled into individual contigs using Sequencher v4.5 and then aligned with the *S. diplocostata* EST contig using ClustalX v2.0.9. Alignment of the EST and RT-PCR derived sequences was used to determine the mRNA coding sequence. These sequences were deposited in the EMBL/Genbank database under the accession numbers FR822186 (gDNA) and FR822187 (mRNA). Alignment of the coding sequence with the gDNA PCR-derived sequence found the presence of a 236bp stretch present in the gDNA sequence but not the mRNA sequence. This was taken to represent an intron. The translated coding sequence was analyzed with InterProScan to search for nitrile hydratase protein domains.

### Phylogenetic Analysis

Four protein sequence alignments were generated; beta-alpha subunit fusion sequences (eukaryotes only), beta subunit sequences (eukaryotes and prokaryotes), alpha subunit sequences (eukaryotes and prokaryotes) and a concatenated alignment of all eukaryotic and prokaryotic sequences. For the concatenated alignment, where alpha and beta subunits were coded by separate genes these sequences were concatenated to generate an ‘artificial’ beta-alpha subunit. In eukaryotic species where only one subunit was identified only that subunit was added to the alignment, since Maximum Likelihood-based phylogenetic methods can account for ‘missing’ data in an alignment. For *P. polycephalum* only the beta subunit was used because of the incomplete alpha subunit data. In generating the single subunit alignments, fusion gene sequences were split into their subunit domains. Beta subunits were designated as the sequence from the N-terminal-most residue until the residue before the alpha subunit domain, as detected by HMMPfam (see table S2). Alpha subunits were designated to be from the residue after the end of the beta subunit (as detected by HMMPfam, see table S2) until the C-terminal-most residue.

The prokaryotic sequences that were to be added to the alignments were selected by taking the top BLASTp hits to the *M. brevicollis* nitrile hydratase subunits (designated as above). Representatives were chosen from each of; Actinobacteria, Cyanobacteria, Firmicutes and Proteobacteria. In all cases alpha and beta subunits gave top hits to the same bacterial strains, except in two cases. For *Roseobacter* sp., the corresponding beta subunit of the CCS2 strain appeared truncated and therefore the beta subunit from the SK209-2-6 strain was selected instead. For *P. thermophila*, the alpha subunit gave a much lower E value in the BLASTp search, so a better scoring sequence from *Rhodococcus* sp. was used instead for the individual alpha subunit alignment. Neither the *P. thermophila* nor *Rhodococcus* sp. sequences were used for the concatenated total alignment. The well-researched Fe-type nitrile hydratase proteins from *Pseudomonas chlororaphis* [25] were also added to the analysis. A list of prokaryotic sequences used is located in table S3.

Alignments were generated using ClustalX v2.0.9 using the default settings (gap opening = 10, gap extension = 0.2, delay divergent sequences = 30%) and the Gonnet Series matrix. Alignments were manually refined to reduce the impact of missing data. The stretch of sequence between the alpha and beta subunits in the fusion proteins was left intact, as this region could not be assigned to one subunit over the other. The final beta-alpha fusion sequence alignment contained 483 positions, the alpha alignment 347 positions, the beta alignment 380 positions and the total concatenated alignment 658 positions.

Prottest [50] found that the RT-REV+G+F+I model provided the best fit (under the Akaike Information Criterion) for the beta subunit alignment, LG+G+F for the alpha alignment, LG+G+F+I for beta-alpha alignment and WAG+G+F+I model for the total concatenated alignment. Maximum likelihood analyses were carried out using PhyML [51] and RaxML [52]; with the proportion of invariant sites estimated, and 4 categories of gamma-distributed rates among sites, with the alpha value estimated. The starting trees were generated by BioNJ, with tree improvement using NNI+SPR and optimized topology and branch lengths. One thousand bootstrapped data sets were analyzed using the same model for each PhyML analysis. One hundred bootstrapped data sets were analyzed using the same model for each RaxML analysis. Trees generated were viewed with FigTree v1.3.1 (Andrew Rambaut, Institute of Evolutionary Biology, University of Edinburgh 2006–2009).

## Supporting Information

Figure S1 **Phylogenetic Tree of alpha subunit proteins.** The tree was produced using maximum likelihood with the LG+G+F+I model from an alignment of 483 positions. Numbers at nodes are bootstrap support percentages from PhyML (1000 replicates)/RaxML (100 replicates). Bootstrap values <70 are shown as *. The scale bar indicates the average number of amino acid substitutions per site. Eukaryotic Key: Opisthokonta = blue SAR = red CCTH = grey Archaeplastida = Green. Prokaryotic Key: Actinobacteria = Magenta Cyanobacteria = Light Green Firmicutes = Purple Proteobacteria = Orange.
(TIF)

Figure S2 **Phylogenetic Tree** of **beta subunit proteins.** The tree was produced using maximum likelihood with the RT-REV+G+F+I model from an alignment of 380 positions. Numbers at nodes are bootstrap support percentages from PhyML (1000 replicates)/RaxML (100 replicates). Bootstrap values <70 are shown as *. The scale bar indicates the average number of amino acid substitutions per site. Eukaryotic Key: Opisthokonta = blue SAR = red CCTH = grey Amoebozoa = brown Archaeplastida = Green. Prokaryotic Key: Actinobacteria = Magenta Cyanobacteria = Light Green Firmicutes = Purple Proteobacteria = Orange.
(TIF)

Figure S3 **Phylogenetic Tree of beta-alpha subunit fusion proteins.** The tree was produced using maximum likelihood with the LG+G+F model from an alignment of 347 positions. Numbers at nodes are bootstrap support percentages from PhyML (1000 replicates)/RaxML (100 replicates). The scale bar indicates the average number of amino acid substitutions per site. Key: Opisthokonta = blue, SAR = red CCTH = grey.
(TIF)

Table S1 **List of taxa, and genomic and transcriptomic sequence databases searched, not including EBML/Genbank dbEST and non-redundant protein and nucleotide data.** In each case, searches were conducted using the *M. brevicollis* nitrile hydratase protein sequence with tBLASTn (vs. nucleotide databases) and/or BLASTp (vs. protein databases) on default settings.
(XLS)

Table S2 **Results of BLAST searches and InterProScan analyses.** All BLAST searches were carried out using the *M. brevicollis* nitrile hydratase protein sequence. Only hits with an E value better than 1E^−05^ are listed. Protein sequences for InterProScan analyses were generated according to the method detailed in the text. In all cases the HMMPfam domain database detected all nitrile hydratase subunits that were present. The numbers in square brackets list the residues the domain signatures are found between.
(DOC)

Table S3 **Prokaryotic nitrile hydratases used in the phylogenetic analyses.** Sequences were chosen from the top BLASTp hits to the *Monosiga brevicollis* nitrile hydratase protein sequence from the firmicute, actinobacterial, cyanobacterial and proteobacterial clades. These sequences were concatenated in a 5′-beta-alpha-3′ arrangement for the total concatenated nitrile hydratase alignment that produced the phylogeny in figure 2. The two subunits from the different *Roseobacter* strains were concatenated together. The subunit sequences from *Rhodococcus* sp. and *Pseudonocardia thermophila* were not used in the concatenated alignment. All alpha subunits and beta subunits were used for the alignments that produced the maximum likelihood trees in figures S1 and S2 respectively.
(XLS)
