# Supplementary figures and images for: Nitrile Hydratase Genes Are Present in Multiple Eukaryotic Supergroups
Source: PLoS One. 2012 Apr 10;7(4):e32867. doi: 10.1371/journal.pone.0032867 (PMC3323583; doi:10.1371/journal.pone.0032867)

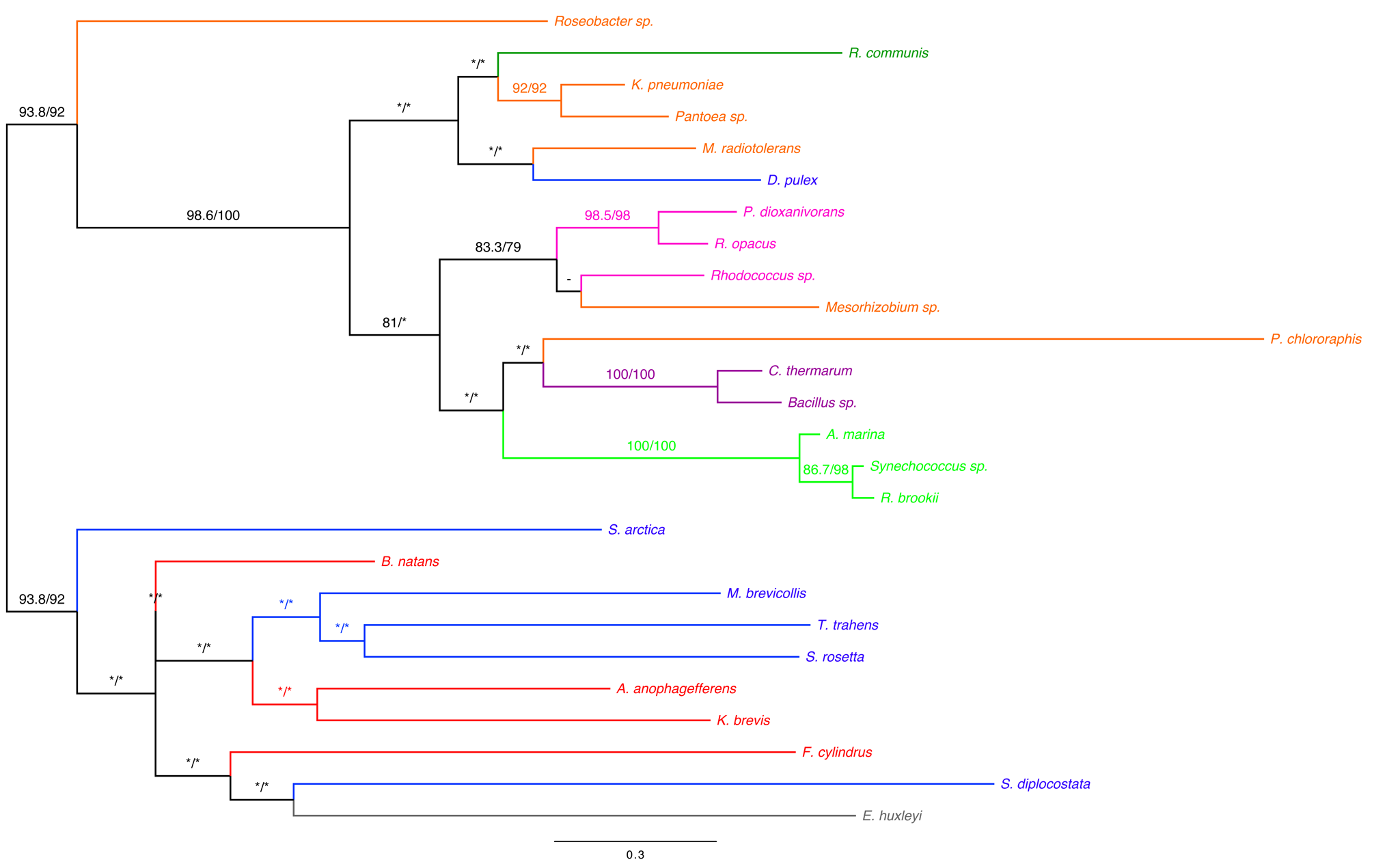

Supplement: Figure S1 — Phylogenetic Tree of alpha subunit proteins. The tree was produced using maximum likelihood with the LG+G+F+I model from an alignment of 483 positions. Numbers at nodes are bootstrap support percentages from PhyML (1000 replicates)/RaxML (100 replicates). Bootstrap values <70 are shown as *. The scale bar indicates the average number of amino acid substitutions per site. Eukaryotic Key: Opisthokonta = blue SAR = red CCTH = grey Archaeplastida = Green. Prokaryotic Key: Actinobacteria = Magenta Cyanobacteria = Light Green Firmicutes = Purple Proteobacteria = Orange. (TIF) [file pone.0032867.s001.tif]

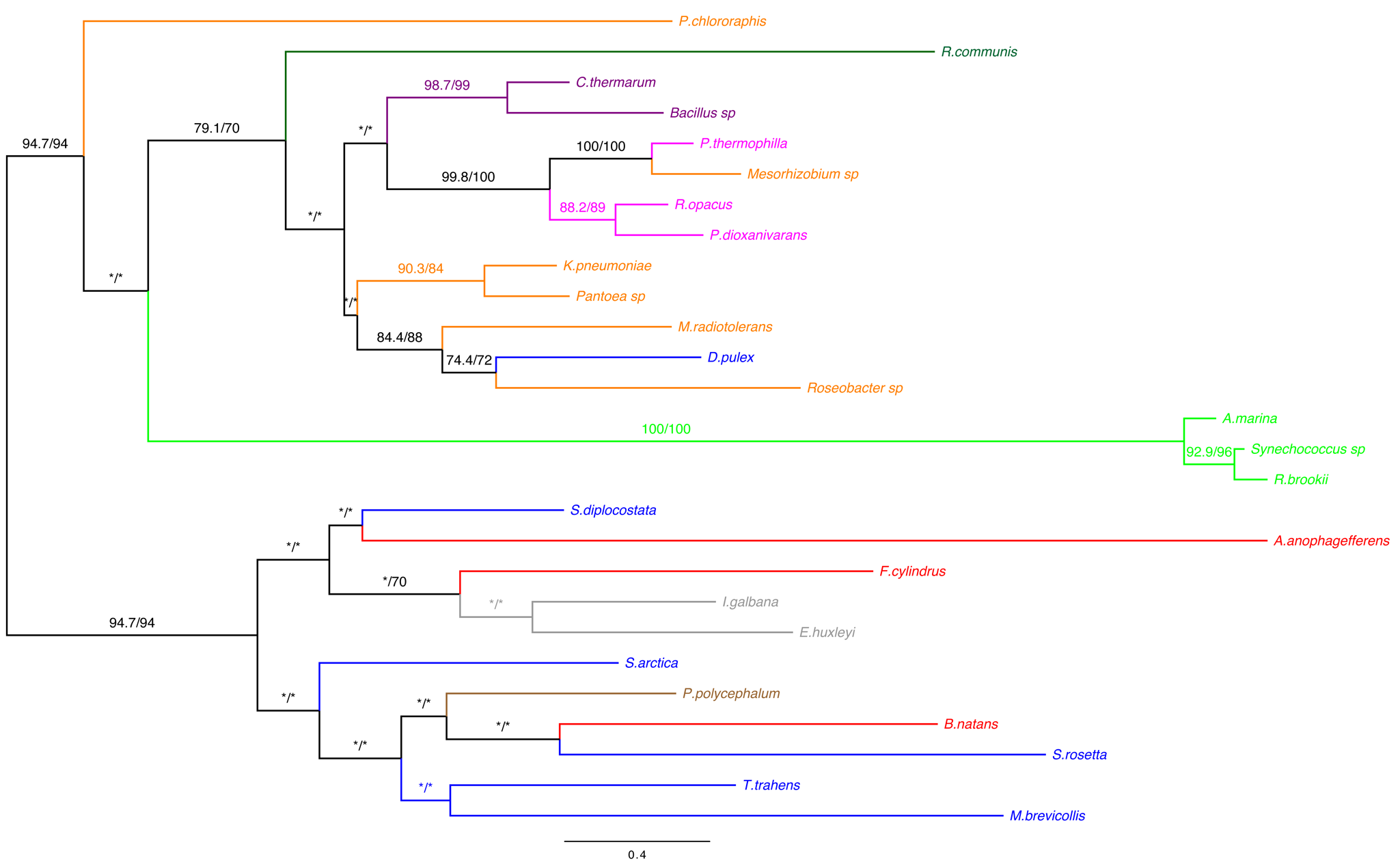

Supplement: Figure S2 — Phylogenetic Tree of beta subunit proteins. The tree was produced using maximum likelihood with the RT-REV+G+F+I model from an alignment of 380 positions. Numbers at nodes are bootstrap support percentages from PhyML (1000 replicates)/RaxML (100 replicates). Bootstrap values <70 are shown as *. The scale bar indicates the average number of amino acid substitutions per site. Eukaryotic Key: Opisthokonta = blue SAR = red CCTH = grey Amoebozoa = brown Archaeplastida = Green. Prokaryotic Key: Actinobacteria = Magenta Cyanobacteria = Light Green Firmicutes = Purple Proteobacteria = Orange. (TIF) [file pone.0032867.s002.tif]

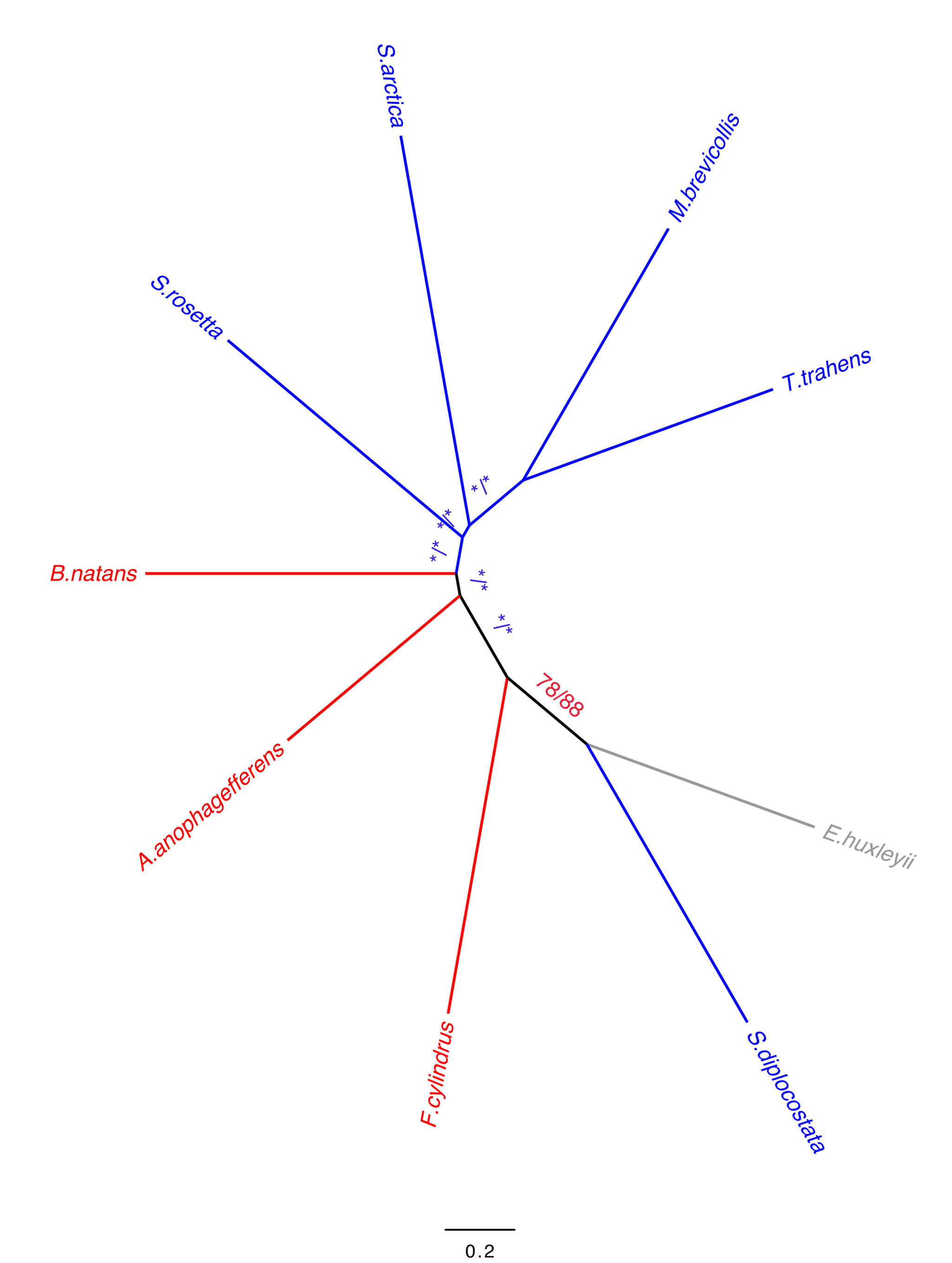

Supplement: Figure S3 — Phylogenetic Tree of beta-alpha subunit fusion proteins. The tree was produced using maximum likelihood with the LG+G+F model from an alignment of 347 positions. Numbers at nodes are bootstrap support percentages from PhyML (1000 replicates)/RaxML (100 replicates). The scale bar indicates the average number of amino acid substitutions per site. Key: Opisthokonta = blue, SAR = red CCTH = grey. (TIF) [file pone.0032867.s003.tif]
