# Supplementary material for: Nitrile Hydratase Genes Are Present in Multiple Eukaryotic Supergroups
Source: PLoS One. 2012 Apr 10;7(4):e32867. doi: 10.1371/journal.pone.0032867 (PMC3323583; doi:10.1371/journal.pone.0032867)
Supplement: Table S2 — Results of BLAST searches and InterProScan analyses. All BLAST searches were carried out using the M. brevicollis nitrile hydratase protein sequence. Only hits with an E value better than 1E−05 are listed. Protein sequences for InterProScan analyses were generated according to the method detailed in the text. In all cases the HMMPfam domain database detected all nitrile hydratase subunits that were present. The numbers in square brackets list the residues the domain signatures are found between. (DOC) [file pone.0032867.s005.doc]

| **Supergroup** | **Clade** | **Species** | **Database Source** | **Accession Number** | **E-value for tBLASTn** | **E-value for Blastp** | **Interproscan HMMPfam Protein Domains** |
| --- | --- | --- | --- | --- | --- | --- | --- |
| Opisthokont | Choanoflagellates | *Monosiga brevicollis* | EMBL/Genbank | GI:167524980 | 0 | 0 | NHase_beta [8-97] 1.9e-13 [107-209] 1.1e-15 NHase_alpha [294-493] 2.1e-64 |
| Opisthokont | Choanoflagellates | *Salpingoeca rosetta* | Broad Institute Origins of Multicelluarity | PTSG_03349 / GI:326437118 | 3.00E-24 | 9.00E-75 | NHase_beta [43-145] 8.2e-12 NHase_alpha [206-408] 8.4e-65 |
| Opisthokont | Choanoflagellates | *Stephanoeca diplocostata* | RT-PCR amplification | FR822187 (unreleased) | 2.00E-22 | n/a | NHase_beta [1-39] 4.5e-05 [50-150] 8e-14 NHase_alpha213-314] 1.3e-28 |
| Opisthokont | Apusozoans | *Thecomonas trahens* | Broad Institute Origins of Multicelluarity | *T. trahens* ATCC 50062: AMSG_07929 | 9.00E-76 | 2.00E-76 | NHase_beta [72-140] 3.7e-11 [156-251] 5.2e-12 NHase_alpha [324-514] 4.5e-68 |
| Opisthokont | Ichthyosporea | *Sphaeroforma arctica* | Broad Institute Origins of Multicelluarity | SARC_06007.1 hypothetical protein (Transcript:SARC_06007T0) | 2.00E-15 | 9.00E-67 | NHase_beta [24-117] 1.8e-11 [128-223] 1.2e-13 NHase_alpha [294-474] 2e-68 |
| Amoebozoan | Myxogastrids | *Physarum polycephalum* | tbESTdb | PPE00007773 | n/a | 8.00E-32 | NHase_beta [2-90] 2e-14 [95-193] 2e-16 |
| Amoebozoan | Myxogastrids | *Physarum polycephalum* | The Genome Institute | Contig3175 14212 17443 | n/a | 3.40E-32 | NHase_alpha [3104-3133] 4.4e-07 [3202-3251] 7.8e-15 |
| SAR (Stramenopile) | Diatoms | *Fragilariopsis cylindrus* | JGI Genome Database | jgi|Fracy1|162296|gw1.15.507.1 | 6.45E-58 | 1.24E-42 | NHase_beta [82-307] 8.1e-39 NHase_alpha [348-529] 1.5e-69 |
| SAR (Stramenopile) | Pelagophytes | *Aureococcus anophagefferens* | JGI Genome Database | Scaffold 7 start 2143143- end 2144648 | 1.33E-75 | 1.87E-57 (alpha only) | NHase_beta [85-172] 1e-12 [176-243] 2e-05 NHase_alpha [284-467] 2.5e-68 |
| CCTH (Haptophyte) | Prymnesiophytes | *Isochrysis galbana* | tbESTdb | ISE00002255 | n/a | 2.00E-05 | NHase_beta [18-86] 5.4e-09 |
| CCTH (Haptophyte) | Prymnesiophytes | *Emiliania huxleyi* | JGI Genome Database | jgi|Emihu1|449479|estExtDG_Genemark1.C_170079 | 5.50E-58 | 5.52E-56 | NHase_beta[34-122] 2.3e-10 [132-230] 3.5e-15  NHase_alpha [302-413] 1.4e-31 [414-457] 1.2e-16 |
| SAR (Alveolate) | Dinoflagellates | *Karenia brevis* | EMBL/Genbank | EX975975.1 GI:159019698 | n/a | 3.00E-55 | NHase_alpha [5-188] 1.5e-66 |
| SAR (Rhizarian) | Chlorarachniophytes | *Bigelowiella natans* | JGI Genome Database | jgi|Bigna1|69681|fgenesh1_pg.9_#_276 | 3.24E-78 | 2.18E-78 | NHase_beta [87-151] 1.1e-8 [159-257] 3.2e-14  NHase_alpha [1-142] 3.8e-68 |
| Archaeplastid | Embryophyte | *Ricinus communis* | EMBL/Genbank | GI:255589101 | 3.00E-41 | 1.00E-46 | NHase_alpha [24-206] 1.8e-89 |
| Archaeplastid | Embryophyte | *Ricinus communis* | EMBL/Genbank | GI:255589104 | 3.00E-08 | 3.00E-06 | NHase_beta [63-189] 2.6e-40 |
| Opisthokont | Metazoan | *Daphnia pulex* | wFleaBase v2010 | hxNCBI_GNO_63564 (scaffold 7175) | 4.00E-34 | 6.00E-33 | NHase_alpha [2-184] 5e-91 |
| Opisthokont | Metazoan | *Daphnia pulex* | wFleaBase v2010 | hxNCBI_GNO_17224 (scaffold 1130) | 2.00E-12 | 1.00E-13 | NHase_beta [1-216] 3.5e-74 |
| Opisthokont | Metazoan | *Danio rerio* | EMBL/Genbank | GI:149695090 | 2.00E-33 | n/a | n/a |
| Archaeplastid | Embryophyte | *Triticum aestivum* | EMBL/Genbank | GI:143432829 | 1.00E-41 | n/a | n/a |
